# A Robust Tracking Method for Multiple Moving Targets Based on Equivalent Magnetic Force

**DOI:** 10.3390/mi13112018

**Published:** 2022-11-18

**Authors:** Ying Wang, Qiang Fu, Yangyi Sui

**Affiliations:** Key Laboratory of Geo-Exploration Instruments, Ministry of Education of China, College of Instrumentation and Electrical Engineering, Jilin University, Changchun 130026, China

**Keywords:** equivalent magnetic force, multiple moving magnetic targets, robust tracking

## Abstract

A ferromagnetic vehicle, such as a submarine, magnetized by the Earth’s magnetic field produces a magnetic anomaly field, and the tracking of moving targets can be realized through real-time analysis of magnetic data. At present, there are few tracking methods based on magnetic field vectors and their gradient tensor. In this paper, the magnetic field vector and its gradient tensor are used to calculate equivalent magnetic force. It shows the direction of the vector between the detector and the tracking targets for controlling the direction of motion of the detector and achieving the purpose of tracking. Compared with existing positioning methods, the proposed method is relatively less affected by instrument resolution and noise and maintains robustness when the velocity vectors of multiple magnetic targets change randomly.

## 1. Introduction

A magnetometer in geophysical applications is a precision instrument used to observe magnetic anomalies caused by rocks, ores, and ferromagnetic objects (such as submarines) which are magnetized by the geomagnetic field [1,2,3]. It is the basis for studying the geological structure, mineral resource distribution, and magnetic characteristics of ferromagnetic objects. With the development of modern physics, many institutions have developed magnetometers based on proton precession, fluxgate, optical pump, and superconductivity [4,5,6,7], and the accuracy of magnetic measurement has continuously improved. According to physical quantities to measure, magnetometers can be divided into three categories: total field magnetometers for the magnitude of the magnetic field, vector magnetometers for three components of the magnetic field [8,9], and tensor magnetometers for magnetic gradient tensor [10,11]. Among them, the magnetic gradient tensor provides rich information and suppresses time-domain interference of the geomagnetic field. It is especially suitable for measurement on mobile platforms and has become an international research frontier [3,10].

An essential application of magnetometers is the real-time tracking of magnetic targets [12]. The current development trend mainly has two aspects: firstly, the magnetic targets to be tracked are more and more abundant, and their motion states are more and more complex [13,14]; secondly, the tracking methods have gradually developed from the initial use of the total field magnetometer to the combination of multiple magnetometers [15,16], such as the combination of vector magnetometer and tensor magnetometer. This is because the total field magnetometer can only recognize the targets’ existence within its detection range through changing magnetic field value from the magnetic anomaly, but it cannot track moving targets due to lack of directivity [17,18]. For example, the responsive search submarine work is to establish a specific search model based on the total field magnetometer or other instruments in a special search scene, and after determining the search range, improve the submersible search efficiency by studying different routes of the detector or other environment-affected parameters [12,19,20,21,22,23]. The combined tracking method of vector magnetometer and tensor magnetometer mainly adopts Euler deconvolution [15,24]. The traditional Euler deconvolution method mainly establishes a linear equation system about the position vector through the potential field relationship between the magnetic field and its gradient tensor. However, there is a problem with numerical calculation stability in tensor inversion. Nara et al. (2014) have pointed out that when the magnetic moment direction is perpendicular to the position vector, the magnetic gradient tensor matrix becomes a singular matrix [25]. This situation is more likely to occur where multiple magnetic targets coexist, with more errors involved in solving results [26]. To better ensure the stability of inversion, Nara et al. have proposed the Euler deconvolution method based on generalized inversion, which has partially solved the numerical stability problem [25]. Yin et al. (2020) have re-analyzed the relationship among position vector, magnetic field vector, and magnetic gradient tensor to avoid inversion calculation of the magnetic gradient tensor [27], but it is still affected by magnetic field noise.

The goal of this paper is the real-time tracking of moving targets in multi-target fields. To that end, we offer an analysis method based on the equivalent magnetic force that can successfully track moving targets in such fields. The tracking performance is inevitably impacted by various noises during motion tracking, particularly the inaccuracy of magnetic field measurement while the detector is moving. The equivalent magnetic force method is more advantageous than the currently employed positioning methods. Because of its high robustness, ease of use, and calculation stability, it can effectively weaken the impact of various noise and measurement errors in the tracking process and guarantee the real-time performance of motion tracking. Additionally, where multiple magnetic targets exist simultaneously, this method can accurately track the moving target with the highest equivalent magnetic force.

## 2. Materials and Methods

### 2.1. Introduction to Tracking Models

**Figure 1:**
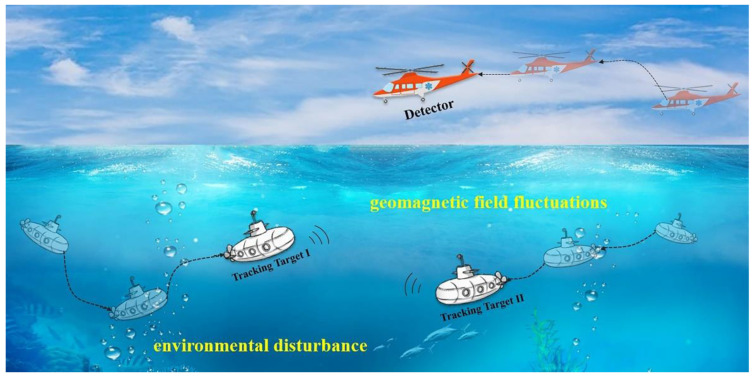
Schematic diagram.

When a magnetic target and a detector are both moving continuously, motion tracking is the process of detecting and analyzing real-time changes in the target’s magnetic field and immediately adjusting the detector’s motion direction. Typically, the magnetic detector keeps a large distance from the tracked target and may not be in the same motion plane. This paper uses a submarine with magnetic properties as an example for detection and tracking to simplify the motion tracking model, as shown in Figure 1.

It is possible to generate independent random motion trajectories by positioning multiple moving targets in the same plane and allowing them to move independently. The detector performs motion tracking in a plane higher than the moving target, so the target is equivalent to a moving magnetic dipole [28]. The measured magnetic field vector and magnetic gradient tensor data change in real-time due to the changing magnetic field as the target moves. The orientation information of the magnetic target and the direction vector of the detector at the next moment can be obtained by analyzing and processing the detection data at the current moment. The detector adjusts its motion direction to the direction vector toward the target. 

Practically speaking, the magnetic anomaly is small in comparison to the total geomagnetic field; therefore, the motion platform’s magnetic field vector is easily influenced by geomagnetic field fluctuations, leading to data inaccuracy. Additionally, the noise of the moving platform and other environmental interference makes tracking more difficult and affects the accuracy of computation results. As a result, a data analysis method that is less influenced by error interference and has high robustness is required.

In the tracking process, it can be considered that the target’s initial position is randomly distributed within a specific range. The target has a maximum speed $v_{\max}$ and its initial speed follows a uniform distribution on $\left[0,v_{\max}\right]$. The heading θ of the target follows a uniform distribution on $\left[-\frac{\eta }{2},\frac{\eta }{2}\right]$ (where η⩽2π) [17].

We equivalent the motion of the magnetic target into the following four models:

Model 1: The heading and the speed remain constant during the movement of the magnetic target.
Model 2: The heading remains constant, and the speed changes randomly during the movement of the magnetic target.
Model 3: The heading changes randomly, and the speed remains constant during the movement of the magnetic target.
Model 4: The heading and the speed change randomly during the movement of the magnetic target.

Convert the coordinate system to a Cartesian coordinate system and keep it constant throughout the detection process. Assume the speed of the detector to be $v_{\max}$, which is the maximum speed of the target, and the sampling interval to be $t_{0}$. The direction vector ***s*** of the detector at the next moment is (L,M,N) in three dimensions. The detector’s displacements in *x* and *y* directions in an interval $t_{0}$ correspond to ΔX and ΔY, respectively, shown in Equations (1) and (2):

(1) $$\Delta X=\frac{Lv_{\max}t_{0}}{\sqrt{L^{2}+M^{2}}}$$

(2) $$\Delta Y=\frac{Mv_{\max}t_{0}}{\sqrt{\begin{array}{c} L^{2}+M^{2} \end{array}}}$$

The detector tracks the moving target in the *XOY* plane. Assume the initial position of the detector to be $\left(X_{0},Y_{0},Z_{0}\right)$ and the magnetic target to be $\left(x_{0},y_{0},z_{0}\right)$. Then at the next moment $t_{1}$, the former will become $\left(X_{1},Y_{1},Z_{1}\right)=\left(X_{0}+\Delta X,Y_{0}+\Delta Y,Z\right)$, and the latter will become $\left(x_{1},y_{1},z_{1}\right)$. At the same time, the relative position relationship between the detector and the moving target has changed. At the next moment $t_{2}$, the direction vector ***s*** of the detector will be recalculated, and the above process will be repeated. Finally, the detector realizes the motion-tracking function in the iterative calculation process.

### 2.2. Introduction to Tracking Methods

From Ampere’s Law, the elementary force on a current element *Id**I*** in the presence of a magnetic field ***B*** is given by dF=I(dI×B) [29]. We can further organize the formula to obtain Equation (3) [30]:

(3) $$F=\langle [\left(M_{r}+\frac{\chi }{\mu_{0}}B\right)\cdot \nabla ]B\rangle V$$

where $M_{r}$ is the remanent magnetic moment, $\mu_{0}$ is the magnetic permeability, χ is the magnetic susceptibility of the test mass, and ***V*** is the volume of the test mass.

When in the presence of a magnetic force sensor, Equation (3) can be used to calculate the precise magnetic force of the magnetic material. Otherwise, when only the magnetic field vector and the magnetic gradient tensor are known, and the target’s magnetic properties are unknown, Equation (4) can be used to calculate the equivalent magnetic force instead.

(4) F=G⋅B

where $B=\left[B_{x};B_{y};B_{z}\right]$ is the magnetic field vector, the vector $F=\left[F_{x};F_{y};F_{z}\right]$ represents the equivalent magnetic force, and Equations (5) and (6) show its components in the *x* direction and the *y* direction, respectively.

(5) $$F_{x}=\nabla B_{x}\cdot B$$

(6) $$F_{y}=\nabla B_{y}\cdot B$$

The magnetic field formed by a magnetic dipole at a single observation point can be expressed by Equation (7).

(7) $$B\left(r,M\right)=\frac{\mu }{4\pi }\frac{3\left(M\cdot r\right)r}{r^{5}}-\frac{M}{r^{3}}\;=\frac{\mu }{4\pi }\frac{3\left(M\cdot r\right)\left(r_{x}i+r_{y}j+r_{z}k\right)-r^{2}\left(M_{x}i+M_{y}j+M_{z}k\right)}{r^{5}}\;=\frac{\mu }{4\pi }\left\{\begin{array}{c} \frac{3\left(M_{x}r_{x}+M_{y}r_{y}+M_{z}r_{z}\right)r_{x}-\left(r_{x}^{2}+r_{y}^{2}+r_{z}^{2}\right)M_{x}}{r^{5}}i+\\ \frac{3\left(M_{x}r_{x}+M_{y}r_{y}+M_{z}r_{z}\right)r_{y}-\left(r_{x}^{2}+r_{y}^{2}+r_{z}^{2}\right)M_{y}}{r^{5}}j+\\ \frac{3\left(M_{x}r_{x}+M_{y}r_{y}+M_{z}r_{z}\right)r_{z}-\left(r_{x}^{2}+r_{y}^{2}+r_{z}^{2}\right)M_{z}}{r^{5}}k \end{array}\right\}$$

where ***M*** is the magnetic moment of the magnetic dipole, ***r*** is the source-to-sensor position vector, μ is the air magnetic permeability, ***i***, ***j***, ***k*** = 1, 2, 3 represent ***x***, ***y***, and ***z*** in the Cartesian coordinate system. 

The magnetic gradient tensor ***G*** is defined as the gradient of the magnetic field vector ***B*** [31], which has nine components and can be represented by the following matrix.

(8) $$G=\nabla B=\left[\nabla B_{x};\;\nabla B_{y};\;\nabla B_{z}\right]=\left[\begin{array}{ccc} \frac{\partial B_{x}}{\partial x} & \frac{\partial B_{x}}{\partial y} & \frac{\partial B_{x}}{\partial z}\\ \frac{\partial B_{y}}{\partial x} & \frac{\partial B_{y}}{\partial y} & \frac{\partial B_{y}}{\partial z}\\ \frac{\partial B_{z}}{\partial x} & \frac{\partial B_{z}}{\partial y} & \frac{\partial B_{z}}{\partial z} \end{array}\right]$$

The magnetic gradient tensor ***G*** can be expressed by the scaled moments and direction tensor [32], each component of which can be represented by Equation (9). Similarly, each component of the magnetic field vector ***B*** in Equation (7) can be represented by Equation (10).

(9) $$G_{jk}=\frac{\partial B_{k}}{\partial x_{j}}=-\frac{\mu }{4\pi }\underset{i}{\sum }M^{B}{}_{i}N^{B}{}_{ijk}$$

(10) $$B_{j}=\frac{\mu }{4\pi }\underset{i}{\sum }M^{B}{}_{i}N^{B}{}_{ij}$$

where $M^{B}{}_{i}=\frac{m_{i}}{r^{3}}$ and $M^{G}{}_{i}=\frac{3m_{i}}{r^{4}}$ are the components of $M^{B}$ and $M^{G}$ respectively, $M^{B}$ represents the scaled moments of ***B***, and $M^{G}$ represents the scaled moments of ***G***. $N^{B}{}_{ij}=3n_{i}n_{j}$-$\delta_{ij}$ and $N^{G}{}_{ijk}=5n_{i}n_{j}n_{k}-\left(\delta_{ki}n_{j}+\delta_{kj}n_{i}+\delta_{ij}n_{k}\right)$ are the components of the direction tensors $N^{B}$ and $N^{G}$, respectively, where $N^{B}$ is a second-order tensor and $N^{G}$ is a third-order tensor. Both $N^{B}$ and $N^{G}$ are functions of the direction cosine $\left(n_{1},n_{2},n_{3}\right)$ of the position vector ***r***. $\delta_{ij}=\{\begin{array}{cc} 1 & i=j\\ 0 & i\neq j \end{array},\;\;n_{1}=\frac{r_{x}}{r},n_{2}=\frac{r_{y}}{r},n_{3}=\frac{r_{z}}{r}$.

Substitute Equations (9) and (10) into Equation (4) and derive the equivalent magnetic force, which is the product of the scaled moments and the orientation tensor as Equation (11).

(11) $$F=G\cdot B\;\;\;\;\;\;\;\;\;\;\;\;\;\;\;\;\;\;\;=N^{G}M^{G}N^{B}M^{B}\;\;\;\;\;\;\;\;\;\;=M^{G}SM^{B}$$

In Equation (11), the principle of tensor analysis [33] is applied to express the equivalent magnetic force as the product of tensors. Then the direction tensor is contracted once to obtain the new direction tensor ***S*** of the equivalent magnetic force ***F***. As shown in Equation (12), ***S*** is also a third-order tensor.

(12) $$S=N^{G}N^{B}={N^{G}}_{ij}g^{i}\overset{⎴}{g^{J}{N^{B}}_{klm}g^{k}g^{l}}g^{m}\;={N^{G}}_{ij}{N^{B}}_{klm}g^{i}g^{k}g^{m}\;=S_{ikm}g^{i}g^{k}g^{m}$$

In this paper, the tracking process is discussed in a Cartesian coordinate system with both base vectors normalized and orthogonal. So when contracting the tensor, it is not necessary to distinguish between the upper and lower indexes of base vectors and the obtained dummy index, so each component $S_{ijk}$ can be expressed as Equation (13):

(13) $$S_{ijk}=N^{G}{}_{ir}N^{B}{}_{jrk}=\left(3n_{i}n_{r}-\delta_{ir}\right)\left[5n_{j}n_{r}n_{k}-\left(\delta_{kj}n_{r}+\delta_{kr}n_{j}+\delta_{jr}n_{k}\right)\right]$$

The direction tensor ***S*** represents the direction of the position vector ***r***. However, due to the objective existence of the magnetic moment direction, the calculated direction vector $s_{F}$ from the equivalent magnetic force method is influenced by $M^{B}$, $M^{G}$, and ***S*** at the same time. The direction vector $s_{F}$ of the detector is obtained after a little deviation, but there is a real-time relative motion relationship between the detector and the moving magnetic source during the motion tracking process, and there is no cumulative error, so it has little impact on the overall tracking effect.

**Figure 2:**
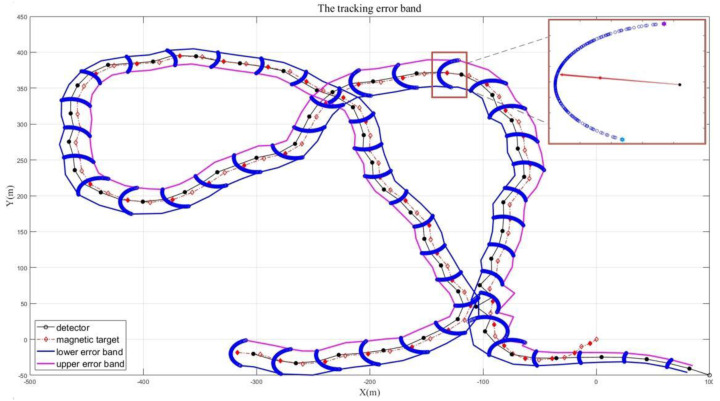
The tracking error band of a single moving target based on the equivalent magnetic force, in which a certain point in the tracking process is partially enlarged to explain the drawing principle.

During the tracking process, the detector will form a tracking trajectory following the moving target. By drawing error bands of the tracking trajectory, the effect of the magnetic moment direction on the direction vector $s_{F}$ is clearly demonstrated. The process and principle of drawing error bands are shown in Figure 2. 

The relative position relationship at a certain moment is used to explain the drawing principle. The blue trace depicts all the positions that the detector might reach at the next moment after being affected by all possible magnetic moment directions. Among these tracking points, respectively find the points that are farthest from the actual pointing (red arrow) vertical distance from the detector to the tracking target. Both sides are the upper and lower error band sample points. As shown in Figure 2, the black dots are the positions of the detector, and the red dots are the positions of the tracking target. It can be observed that the tracking trajectories generated by the equivalent magnetic force analysis method are always within the drawn error band.

## 3. Results

### 3.1. Motion Tracking Simulation

**Figure 3:**
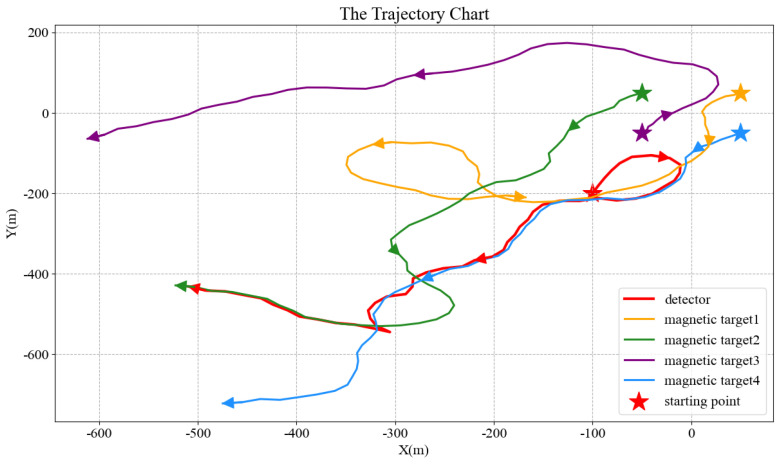
Real−time motion tracking of multiple magnetic targets, in which the motion tracking time *t* = 50 s, and the sampling interval $t_{0}$ = 1 s.

Each moving target is considered a separate magnetic submarine, which generates random trajectories according to the motion models. The initial positions of the four targets are (50, 50), (−50, 50), (−50, −50), and (50, −50), with *z* = 0, and they individually move according to Model 4. The initial position of the detector is set to (−100, −200, −50). When the tracking time *t* = 50 s and the sampling interval $t_{0}$ = 1 s, we obtain the result shown in Figure 3.

Among all the moving targets, the direction vector points to the one with the greatest equivalent magnetic force or the closest relative distance from the detector, which is thus tracked in the subsequent process. We set four moving targets with the same magnetic field intensity, as shown in Figure 3. In the initial position, target 3 has both the closest relative distance and the greatest equivalent magnetic force compared with others. Therefore, at the early stage of tracking, the detector will move towards target 3 according to the direction vector $s_{F}$. However, as the tracking progresses, the equivalent magnetic force of target 4 gradually becomes the greatest, and at a specific time, the next target changes into target 4. The motion trajectories of different targets can occasionally cross because they move in completely distinct ways. Since the detector always tracks the moving target with the highest equivalent magnetic force, the tracking target is not lost. As shown in Figure 3, when the motion trajectories of target 2 and target 4 crossed, the tracking target of the detector switched from target 4 to target 2.

### 3.2. Robustness Analysis

#### 3.2.1. Comparative Analysis of Noise Interference

Nara et al. (2006) have demonstrated the principal formula of Euler’s deconvolution [15].

(14) $$r=-3G^{-1}\cdot B$$

In the calculation process, the inverse of the magnetic gradient tensor ***G*** needs to be solved.

When the magnetic moment vector ***M*** is perpendicular to the position vector ***r***, the magnetic gradient tensor matrix changes into a singular matrix, and the condition number approaches infinity, according to a discovery by Nara et al. [25]. Considering this situation, they proposed the Euler deconvolution method based on Moore–Penrose generalized inverse. When the magnetic gradient tensor matrix is singular, the generalized inverse method can be used to obtain the unique solution of the position vector.

According to Zhang’s theory [34], the condition number is a key index to quantify numerical stability. During motion tracking, the angle between the magnetic moment vector ***M*** and the position vector ***r*** inevitably approaches 90 degrees in some cases. Since the magnetic gradient tensor matrix ***G*** is not singular then, corrections cannot be achieved using the generalized inverse method. In such cases, however, the condition number is relatively large, and the equation for the position vector is pathological, which means that when the initial value of the magnetic field vector ***B*** is slightly disturbed, the solution of the equation changes significantly.

Based on these works, Yin et al. focused on the relationships between the analytical formula and the gradient tensor of the magnetic field vector to avoid the inversion problem associated with traditional Euler deconvolution methods [27]. The non-inverting Euler method proposed by Yin et al. can be organized as Equation (15).

(15) $$r=\frac{3\left(G+\lambda_{med}E\right)B}{\lambda_{\min}\lambda_{\max}}=\frac{3GB}{\lambda_{\min}\lambda_{\max}}+\frac{3\lambda_{med}EB}{\lambda_{\min}\lambda_{\max}}$$

where $\lambda =\left(\lambda_{\min},\lambda_{med},\lambda_{\max}\right)$ represents the eigenvalues of the magnetic gradient tensor matrix ***G*** and ***E*** is a 3×3 unit matrix.

Compared with Equation (4), which represents the equivalent magnetic force method, the Equation (15) still introduces magnetic disturbances through its second term, which further affects the tracking performance in actual situations.

**Figure 4:**
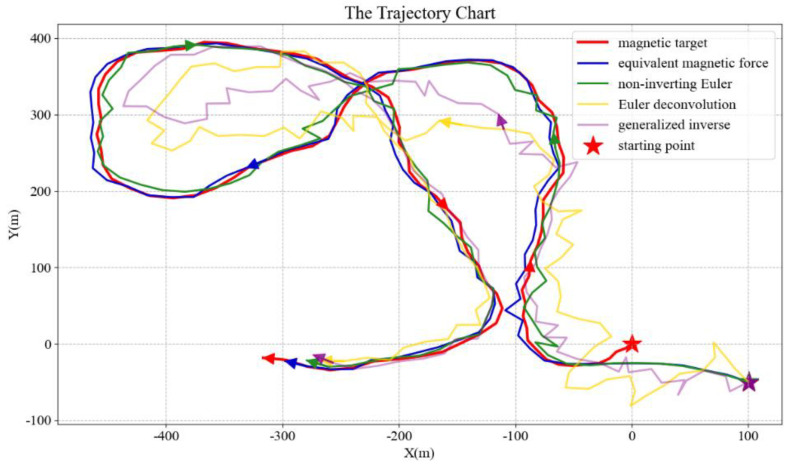
Comparison of tracking performance of various methods. The moving magnetic target generates red tracks, and the other four tracks correspond to the four methods, respectively. The five-pointed star represents the starting point.

The actual tracking process generates a variety of complex motion trajectories. For different methods, their tracking performances, especially in a superimposed noise environment, show a contrast in robustness. The motion tracking time *t* is set to 100 s, and a complex motion trajectory is obtained in the simulation. The magnetic field vector ***B*** and its gradient tensor ***G*** are then superimposed with a Gaussian white noise having a signal-to-noise ratio of 4 and 10, respectively. As seen in Figure 4, when the same noise interferences are superimposed, the tracking performances of the Euler deconvolution method and the generalized inverse method are both significantly impacted, while the equivalent magnetic force method and the non-inverting Euler method perform much better.

**Figure 5:**
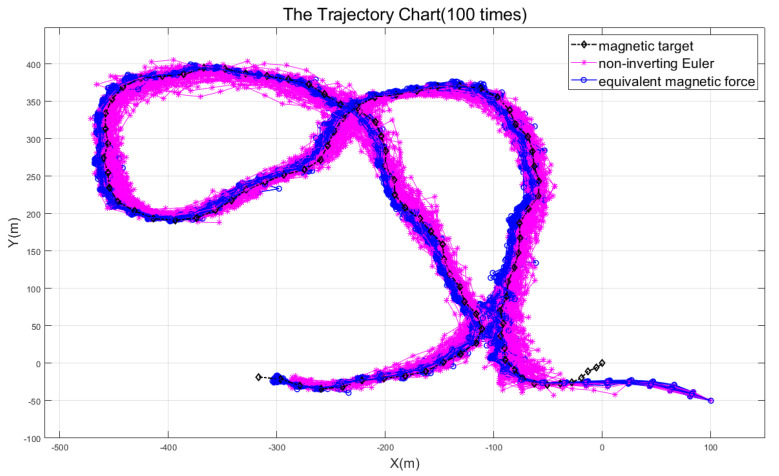
Robustness comparison diagram of the non-inverting Euler method and equivalent magnetic force method.

The numerical calculation process becomes unstable and the tracking effect is not satisfactory since the first two methods require inversion of the magnetic gradient tensor matrix ***G***. In contrast, the latter two methods do not have this problem. However, a careful comparison of these two methods will discover that the robustness of the non-inverting Euler method is still significantly lower than that of the equivalent magnetic force method, as shown in Figure 5.

Keep the noise level and run the simulation 100 times at random. Then the non-inverting Euler method and the equivalent magnetic force method may each provide 100 tracking trajectories. Figure 5 plots all tracking trajectories collectively. By comparison, it is evident that the equivalent magnetic force method is more stable and reliable than the non-inverting Euler method, which further verifies the high robustness of the proposed method in this paper.

**Figure 6:**
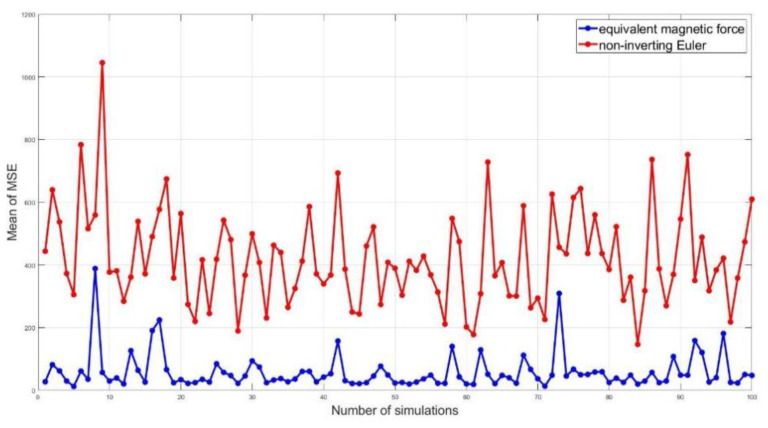
The mean value of the MSE for 100 groups of random trajectory tracking.

The mean square error (MSE) between the motion trajectory and the tracking trajectory is used as an index to evaluate the tracking performance. In order to prove that the equivalent magnetic method has a better tracking performance under any complex trajectory, the non-inverting Euler method is still used as the comparison method. Following Model 4, 100 arbitrary motion trajectories are randomly generated, and the tracking process is repeated 100 times at the same noise level, calculating the MSE each time. As shown in Figure 6, the mean value of the MSE for any complex motion trajectory using the non-inverting Euler method is larger than the equivalent magnetic force method.

#### 3.2.2. Analysis of Multi-Source Coupling Interference

When multiple magnetic targets coexist in the detection area, the overall magnetic field is the superposition of the magnetic fields from all the targets. Assuming that there are two magnetic targets in the detection area, the measured magnetic gradient tensor ***G*** and magnetic field vector ***B*** are, respectively, expressed as Equations (16) and (17) according to the superposition principle. Suppose the eigenvalue of target 1 is $\lambda^{1}=\left(\lambda_{\min}^{1},\lambda_{med}^{1},\lambda_{\max}^{1}\right)$, the eigenvalue of target 2 is $\lambda^{2}=\left(\lambda_{\min}^{2},\lambda_{med}^{2},\lambda_{\max}^{2}\right)$, and the eigenvalue of Equation (16) is $\lambda^{s}=\left(\lambda_{\min}^{s},\lambda_{med}^{s},\lambda_{\max}^{s}\right)$. It is unreasonable to consider $\lambda^{s}=\lambda^{1}+\lambda^{2}$.

(16) $$G=G_{1}+G_{2}=\left[\begin{array}{ccc} G_{xx1} & G_{xy1} & G_{xz1}\\ G_{xy1} & G_{yy1} & G_{yz1}\\ G_{xz1} & G_{yz1} & G_{zz1} \end{array}\right]+\left[\begin{array}{ccc} G_{xx2} & G_{xy2} & G_{xz2}\\ G_{xy2} & G_{yy2} & G_{yz2}\\ G_{xz2} & G_{yz2} & G_{yz2} \end{array}\right]=\left[\begin{array}{ccc} G_{xx1}+G_{xx2} & G_{xy1}+G_{xy2} & G_{xz1}+G_{xz2}\\ G_{xy1}+G_{xy2} & G_{yy1}+G_{yy2} & G_{yz1}+G_{yz2}\\ G_{xz1}+G_{xz2} & G_{yz1}+G_{yz2} & G_{zz1}+G_{yz2} \end{array}\right]$$

(17) $$B=B_{1}+B_{2}=\left[\begin{array}{c} B_{x1}\\ B_{y1}\\ B_{z1} \end{array}\right]+\left[\begin{array}{c} B_{x2}\\ B_{y2}\\ B_{z2} \end{array}\right]=\left[\begin{array}{c} B_{x1}+B_{x2}\\ B_{y1}+B_{y2}\\ B_{z1}+B_{z2} \end{array}\right]$$

The numerical calculation formula of the Euler deconvolution method can be written as Equation (18):

(18) $$r=-3G^{-1}\cdot B=-3\frac{G^{*}}{\left|G\right|}\cdot B$$

Each term in Equation (18) will change accordingly due to the coexistence of multiple magnetic targets, assuming that

(19) $$\begin{array}{c} G^{*}=\left[\begin{array}{ccc} G_{yy}G_{zz}-G_{yz}^{2} & G_{xz}G_{yz}-G_{xy}G_{zz} & G_{xy}G_{yz}-G_{xz}G_{yy}\\ G_{yz}G_{xz}-G_{xy}G_{zz} & G_{xx}G_{zz}-G_{xz}^{2} & G_{xy}G_{xz}-G_{xx}G_{yz}\\ G_{xy}G_{yz}-G_{xz}G_{yy} & G_{xy}G_{xz}-G_{xx}G_{yz} & G_{xx}G_{yy}-G_{xy}^{2} \end{array}\right]\\ =\left[\begin{array}{ccc} G_{11} & G_{12} & G_{13}\\ G_{12} & G_{22} & G_{23}\\ G_{13} & G_{23} & G_{33} \end{array}\right] \end{array}$$

Substitute Equation (19) into Equation (18), and then we can obtain Equation (20).

(20) $$r=\left[\begin{array}{c} r_{x}\\ r_{y}\\ r_{z} \end{array}\right]=\frac{-3}{\left|G\right|}\left[\begin{array}{c} G_{11}B_{x}+G_{12}B_{y}+G_{13}B_{z}\\ G_{12}B_{x}+G_{22}B_{y}+G_{23}B_{z}\\ G_{13}B_{x}+G_{23}B_{y}+G_{33}B_{z} \end{array}\right]$$

In the field where two magnetic targets coexist, according to the superposition principle, each component needs to be adjusted, for example, $G_{11}$ is as shown in Equation (21)

(21) $$G_{11}=\left(G_{yy1}+G_{yy2}\right)\left(G_{zz1}+G_{zz2}\right)-{\left(G_{yz1}+G_{yz2}\right)}^{2}$$

Here we analyze a small polynomial that appears during Euler deconvolution, such as $G_{11}B_{x}$:

(22) $$G_{11}B_{x}=\left[\left(G_{yy1}+G_{yy2}\right)\left(G_{zz1}+G_{zz2}\right)-{\left(G_{yz1}+G_{yz2}\right)}^{2}\right]\left(B_{x1}+B_{x2}\right)\;=\left(G_{yy1}G_{zz1}-G_{yz1}^{2}\right)B_{x1}+\left(G_{yy2}G_{zz2}-G_{yz2}^{2}\right)B_{x2}+\Delta_{r}$$

(23) $$\begin{array}{c} \Delta_{r}=\left(G_{yy1}G_{zz1}-G_{yz1}^{2}\right)B_{x2}+\left(G_{yy2}G_{zz2}-G_{yz2}^{2}\right)B_{x1}+\\ \left(G_{yy1}G_{zz2}+G_{yy2}G_{zz1}-2G_{yz1}G_{yz2}\right)\left(B_{x1}+B_{x2}\right) \end{array}$$

Each item of $\Delta_{r}$ is an interference item caused by the interaction between two targets. The impact will be stronger when there coexist four magnetic targets.

The calculation formula for the equivalent magnetic force method can be written as Equation (24).

(24) $$F=G\cdot B=\left(G_{1}+G_{2}\right)\left(B_{1}+B_{2}\right)$$

Similar to Equation (20), Equation (24) can also be written as follows:

(25) $$F=\left[\begin{array}{c} F_{x}\\ F_{y}\\ F_{z} \end{array}\right]=\left[\begin{array}{c} \nabla B_{x}\cdot B\\ \nabla B_{y}\cdot B\\ \nabla B_{z}\cdot B \end{array}\right]=\left[\begin{array}{c} G_{xx}B_{x}+G_{xy}B_{y}+G_{xz}B_{z}\\ G_{xy}B_{x}+G_{yy}B_{y}+G_{yz}B_{z}\\ G_{xz}B_{x}+G_{yz}B_{y}+G_{zz}B_{z} \end{array}\right]$$

We also analyze a small polynomial in the equivalent force method, such as $G_{xx}B_{x}$:

(26) $$G_{xx}B_{x}=\left(G_{xx1}+G_{xx2}\right)\left(B_{x1}+B_{x2}\right)\;=G_{xx1}B_{x1}+G_{xx2}B_{x2}+\Delta_{f}$$

(27) $$\Delta_{f}=G_{xx1}B_{x2}+G_{xx2}B_{x1}$$

The value in $\Delta_{f}$ corresponds to the interference term caused by the interaction between two magnetic targets. A comparison of Equations (23) and (27) shows that although the equivalent magnetic force method also has interference terms, they are much less than those of the Euler deconvolution method. Note that the influence of interference in |G| has not been considered in this comparison.

Additionally, in multi-target situations, the second term of the non-inverting Euler method is more strongly impacted by the superposition of various error disturbances in the targets’ magnetic field vectors, as shown in Equation (28). Additionally, $\lambda^{s}=\lambda^{1}+\lambda^{2}$ is considered unreasonable when using Equation (15) for calculation. Therefore, the non-inverting Euler method is incorrect when multiple magnetic targets coexist.

(28) $$r=\frac{3GB}{\lambda_{\min}\lambda_{\max}}+\frac{3\lambda_{med}EB}{\lambda_{\min}\lambda_{\max}}\;=\frac{3\left(G_{1}+G_{2}\right)\left(B_{1}+B_{2}\right)}{\lambda_{\min}^{s}\lambda_{\max}^{s}}+\frac{3\lambda_{med}^{s}E\left(B_{1}+B_{2}\right)}{\lambda_{\min}^{s}\lambda_{\max}^{s}}$$

Theoretical analysis can further demonstrate that the equivalent magnetic force method retains excellent robustness in multi-target situations.

## 4. Discussion and Conclusions

The proposed method in this paper can be used to track magnetic targets more accurately after the total field magnetometer determines the existence of magnetic anomalies in the detection range. Remember that if the vertical distance between the detector and the target is set too far apart, the direction of the equivalent magnetic force $s_{F}$ may be primarily biased toward the *z* direction and the direction vector $s_{F}$ may be more easily affected and become inaccurate. This is because both the magnetic moment direction ***M*** and the direction tensor ***S*** have impacts on the direction vector $s_{F}$. For instance, when the magnetic inclination of the detection area is relatively small, the vertical distance above should be reduced to ensure the accuracy of the direction vector $s_{F}$ in the *XOY* plane because the magnetic moment direction has a greater impact on the *x* direction and *y* direction relative to the *z* direction.

In addition, force balancing issues may occur when the detector is affected by multiple targets. However, on the one hand, there are many disturbances in the natural detection environment, so a constant balance is unlikely to exist. On the other hand, due to the influence of the magnetic moment direction vector ***M***, the direction vector $s_{F}$ will deviate from the equilibrium position, further ensuring the robustness of the proposed method.

The method in this paper is based on the direction vector of the equivalent magnetic force, and the numerical solution process is stable. It significantly guarantees high robustness and reduces the effects of noise disturbances, particularly the measurement inaccuracy of the magnetic field vector, on the tracking performance. In future research, we will analyze the numerical relationship of equivalent magnetic forces more accurately and deeply to obtain more information about moving magnetic targets, such as their magnetic magnitude.

## 5. Patents

This section is not mandatory but may be added if there are patents resulting from the work reported in this manuscript.

## Data Availability

This study does not report any data.
